# Risk of breast cancer in women with non-lactational mastitis

**DOI:** 10.1038/s41598-019-52046-3

**Published:** 2019-10-30

**Authors:** Chun-Ming Chang, Mei-Chen Lin, Wen-Yao Yin

**Affiliations:** 1 Department of General Surgery, Hualien Tzu Chi Hospital, Buddhist Tzu Chi Medical Foundation, Hualien, Taiwan; 2 Management Office for Health Data, China Medical University Hospital, Taichung, Taiwan; 3 Department of General Surgery, Dalin Tzu Chi Hospital, Buddhist Tzu Chi Medical Foundation, Chiayi, Taiwan; 4 College of Medicine, Tzu Chi University, Hualien, Taiwan; 5 College of Medicine, China Medical University, Taichung, Taiwan

**Keywords:** Breast cancer, Risk factors

## Abstract

Little is known regarding the association of non-lactational mastitis women with breast cancer risk. This population-based cohort study examined the breast cancer risk in women with non-lactational mastitis. We identified 3,091 women with non-lactational mastitis between 2000 and 2011 using the Taiwan National Health Insurance Research Database. We performed 1:4 propensity score matching by age, socioeconomic status and comorbidities and identified 12,364 women without non-lactational mastitis. The mean age of women with non-lactational mastitis was 37.9 years; these women had a higher breast cancer risk than the comparison group (adjusted hazard ratio = 1.94, 95% confidence interval: 1.30–2.90). The incidence rates of breast cancer in women with non-lactational mastitis and the comparison group were 14.79 and 7.57 per 10,000 person-years, respectively. Furthermore, non-lactational mastitis was a risk factor for breast cancer in women aged <50 years, women with lower socioeconomic status and women with hormonal medication (*p* < 0.05). Women who had more episodes of non-lactational mastitis had a higher risk of developing a breast cancer. Thus, the risk of breast cancer in women with non-lactational mastitis is significantly higher than those without non-lactational mastitis.

## Introduction

Breast cancer is the most commonly diagnosed malignancy, accounting for 1 in 4 cancer cases among women worldwide^1,2^. Treatment for breast cancer has improved, such that early treatment of breast cancer is associated with a good survival rate^3^. It is important to identify women who have a higher risk of breast cancer to ensure that they can take necessary precautionary measures.

Mastitis refers to breast inflammation, comprising lactational and non-lactational mastitis. Lactational mastitis constitutes acute inflammation of the breast in connection with pregnancy or breastfeeding, and it occurs in 2–10% of breastfeeding women^4^. Non-lactational mastitis is a breast inflammatory condition in non-breastfeeding women. The two major entities of non-lactational mastitis are periductal mastitis and idiopathic granulomatous mastitis and both of them primarily affect young women^5–8^.

Little is known regarding the risk of breast cancer in women with non-lactational mastitis. Owing to many studies supporting strong associations between chronic inflammatory conditions and tumorigenesis, a link between inflammation and cancer has been recognized^9–11^. Consequently, various infectious and inflammatory diseases are strongly associated with a high risk of malignancy in the corresponding organs^12–15^. However, whether or not women with non-lactational mastitis, as opposed to those without non-lactational mastitis, have a higher risk of breast cancer remains unknown. Therefore, the aim of this population-based cohort study was to examine the breast cancer risk in women with non-lactational mastitis using a national database of Taiwan. We also evaluated the risk of breast cancer among women with different numbers of episodes of non-lactational mastitis.

## Methods

### Data source

Taiwan National Health Insurance (NHI) provides universal insurance coverage and is a single-payer system with the government as the sole insurer. It covers more than 99% of the Taiwanese population of 23.4 million people and has contracts with 97% of hospitals and clinics in Taiwan. Data regarding registration files and medical claims for all beneficiaries are linked through encrypted identification numbers in the National Health Insurance Research Database (NHIRD) (http://nhird.nhri.org.tw/en/index.htm). All diagnoses in the database are coded in accordance with the International Classification of Disease, Ninth Revision, Clinical Modification (ICD-9-CM).

The National Health Research Institute of Taiwan has randomly selected 1 million NHI beneficiaries to establish a longitudinal database. No statistically significant differences in age, sex and health expenditure were found between the 1 million individuals in the longitudinal database and all NHI beneficiaries. This data set of 1 million randomly selected people was used in this study.

The Research Ethics Committee of China Medical University and Hospital in Taiwan approved the study (IRB permit number: CMUH-104-REC2-115-R3). Because we used the encrypted NHIRD dataset, the IRB determined that informed consent was not needed. Our research was performed in accordance with relevant guidelines and regulations.

### Study population and design

Lactational mastitis in the current study was defined as mastitis that occurred in women within 1 year of giving birth to a baby. Non-lactational mastitis was defined as mastitis that occurred in women beyond 1 year after giving birth to a baby, or in women who had never given birth to a baby.

**Figure 1:**
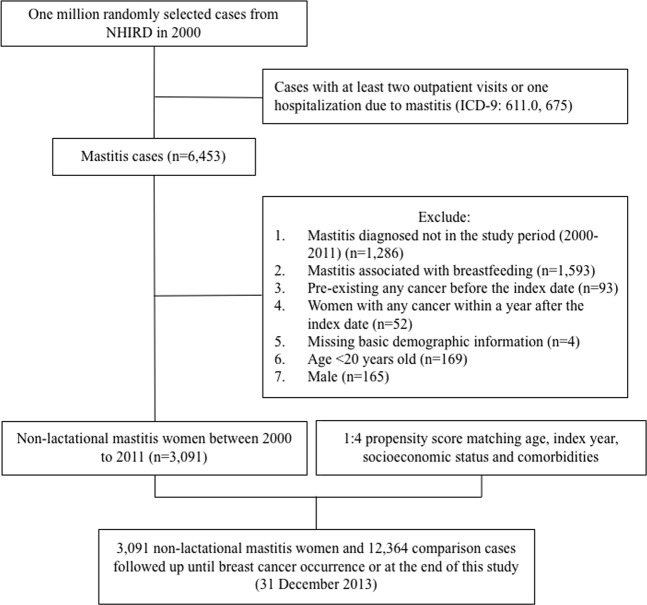
Flowchart for identifying women with non-lactational mastitis and the comparison group within the National Health Insurance Research Database.

Figure 1 shows a flowchart of the method used to generate the non-lactational mastitis and comparison cohorts. First, we used ICD-9 codes (ICD-9: 611.0 and 675) to identify the mastitis group: women in the NHIRD who were newly diagnosed with mastitis or abscess between 2000 and 2011. Women who had at least two outpatient visits or one hospitalisation due to mastitis or abscess were included. The first date of mastitis diagnosis was established as the index date. To generate the non-lactational mastitis cohort, we excluded women with the following characteristics from the mastitis group: women who had lactational mastitis, women who had any pre-existing cancer before the index date, women who had any cancer including breast cancer within 1 year after the index date, women who did not have basic demographic information, women less than 20 years of age and men (Fig. 1, exclusion criteria). Exclusions were made for the following reasons: pre-existing cancer may become a competing mortality risk before the development of breast cancer; therefore, we excluded women who had previous histories of other cancers before the diagnosis of mastitis. In addition, we excluded women with breast cancer diagnosed within 1 year after the index date to avoid misdiagnosis of inflammatory breast cancer. The comparison group included women without any mastitis diagnosis, and the same exclusion criteria were applied. To avoid the influence of baseline differences in terms of age, index year, socioeconomic status (SES) and comorbidities, we performed 1:4 propensity score matching. The two groups were followed up from the index date until the occurrence of breast cancer, withdrawal from the database, or until 31 December 2013.

Comorbidities and hormonal medications were identified using ICD-9 codes from the NHIRD. Comorbidities that have been reported as potential risk factors for breast cancer include schizophrenia^16^, hypertension^17^, chronic obstructive pulmonary disease^18^, thyroid disease^19,20^, diabetes^21^, hyperlipidaemia^22^ and obesity^23^.

We used income-related insurance payment amounts as a proxy measure of individual SES. The SES was classified into four groups: (1) lower than US$528/month (equivalent to NT$15,840/month); (2) between US$528 and US$960/month (NT$15,841 to NT$28,800/month); (3) between US$960 and US$1526/month (NT$28,801 to NT$45,800/month); (4) greater than US$1526/month (NT$45,800/month). We selected US$528/month (NT$15,840/month) as the lowest income level for the SES cut-off point because this was the government-stipulated minimum wage for a full-time employee in Taiwan.

If a woman had more than one occurrence of non-lactational mastitis and the treatment interval was longer than 3 months, the second occurrence was defined as new non-lactational mastitis. We measured the occurrence of non-lactational mastitis per year among women in non-lactational mastitis cohort. We divided this occurrence data into three tertiles and analysed the risk of breast cancer.

We used the same procedure, excluding women with non-lactational mastitis, to generate the lactational mastitis cohort and analysed the breast cancer risk of women with lactational mastitis (Supplementary).

### Statistical analysis

Four control women were matched to each woman with non-lactational mastitis who had a similar propensity score, based on nearest neighbour matching without replacement using calliper width within 0.1^24^. Differences in each variable in the non-lactational mastitis and comparison cohorts were calculated by using the standardised mean difference (SMD)^25,26^; an SMD of <0.1 was considered to indicate a negligible difference^25^. We described the grouped age, comorbidities and medications in the two cohorts as numbers and percentages; we presented the mean ages as means and standard deviations.

To estimate the risk of breast cancer in the non-lactational mastitis and comparison cohorts, hazard ratios (HRs), adjusted hazard ratios (aHRs) and 95% confidence intervals (CIs) were evaluated by using crude and adjusted Cox proportional hazard models, respectively. All statistical analyses were performed using SAS statistical software, version 9.4 (SAS Institute Inc., Cary, NC, USA). The figure for the cumulative incidence curve was plotted by R software. Significance was indicated by a two-sided p-value of <0.05.

## Results

A total of 3,091 women with non-lactational mastitis were enrolled in this study (Table 1). The mean age of the women with non-lactational mastitis was 37.9 years; most (64%) were <40 years of age. After matching by age, SES and comorbidities, the SMDs between the non-lactational mastitis and comparison groups did not significantly differ (SMD <0.1), with the exception of the rate (%) of hormonal medication, which was higher in the non-lactational mastitis group than in the comparison group (SMD = 0.35).

**Table 1:** Demographic characteristics of the non-lactational mastitis women and the comparison group.

	Total	Comparison	Non-lactational Mastitis	Standardized mean difference^§^
	n = 15455	n = 12364	n = 3091	
	n	n (%)/mean (SD)	n (%)/mean (SD)	
Age at baseline^‡^		37.7 (12.5)	37.9 (11.9)	0.021
<40	9748	7767 (62.8)	1981 (64.1)	0.026
40–49	3268	2640 (21.4)	628 (20.3)	0.025
50–59	1559	1246 (10.1)	313 (10.1)	0.002
≥60	880	711 (5.8)	169 (5.5)	0.012
**Monthly income (NT$)**				
0–15840	8563	6856 (55.5)	1707 (55.2)	0.005
15841–28800	5084	4068 (32.9)	1016 (32.9)	0.001
28801–45800	1389	1111 (9)	278 (9)	0.000
>45800	419	329 (2.7)	90 (2.9)	0.015
**Baseline comorbidity**				
Schizophrenia	388	310 (2.5)	78 (2.5)	0.001
Hypertension	1767	1381 (11.2)	386 (12.5)	0.041
Chronic obstructive pulmonary disease	1780	1408 (11.4)	372 (12)	0.020
Thyroid disease	1352	1064 (8.6)	288 (9.3)	0.025
Diabetes	1233	978 (7.9)	255 (8.2)	0.012
Hyperlipidemia	1936	1532 (12.4)	404 (13.1)	0.020
Obesity	201	158 (1.3)	43 (1.4)	0.010
**Medication**				
Hormonal medication	8278	6192 (50.1)	2086 (67.5)	0.359

Abbreviation: SD, standard deviation.

^‡^Student’s t test.

^§^A standardized mean difference of <0.1 indicates a negligible difference between the two cohorts.

Table 2 shows the events, HRs and risk factors of breast cancer. There were a total of 111 cases of newly diagnosed breast cancer. Women with non-lactational mastitis had a significantly higher risk of breast cancer (aHR = 1.94, 95% CI: 1.30–2.90). Age was also an important risk factor for breast cancer: women aged 40–49 years (aHR = 2.60, 95% CI: 1.63–4.14), 50–59 (aHR = 2.67, 95% CI: 1.43–4.96) and women aged >60 years (aHR = 2.66, 95% CI: 1.41–6.21) had higher risks of breast cancer than women aged <40 years.

**Table 2:** Cox proportional hazards regression measured hazard ratio of breast cancer.

	Event	Crude		Adjusted	
	(n = 111)	HR (95% CI)	p-value	HR (95% CI)	p-value
**Non-lactational mastitis**					
No	74	Ref.		Ref.	
Yes	37	1.94 (1.31–2.87)	0.001	1.94 (1.30–2.90)	0.001
**Age at baseline**					
<40	42	Ref.		Ref.	
40–49	42	2.96 (1.93–4.54)	<0.001	2.60 (1.63–4.14)	<0.001
50–59	18	3.04 (1.75–5.28)	<0.001	2.67 (1.43–4.96)	0.002
≥60	9	2.92 (1.42–6.01)	0.004	2.66 (1.14–6.21)	0.023
**Monthly income (NT$)**					
0–15840	38	Ref.		Ref.	
15841–28800	52	2.01 (1.32–3.05)	0.001	1.42 (0.91–2.22)	0.127
28801–45800	14	2.02 (1.09–3.72)	0.025	1.34 (0.71–2.55)	0.370
>45800	7	3.41 (1.52–7.63)	0.003	2.41 (1.05–5.53)	0.038
**Baseline comorbidity**					
Schizophrenia	6	2.46 (1.08–5.6)	0.032	2.66 (1.16–6.09)	0.021
Hypertension	18	1.68 (1.02–2.79)	0.044	0.91 (0.5–1.67)	0.773
Chronic obstructive pulmonary disease	16	1.54 (0.91–2.63)	0.108	1.15 (0.66–2.00)	0.611
Thyroid disease	6	0.71 (0.31–1.62)	0.416	0.58 (0.25–1.32)	0.194
Diabetes	13	1.75 (0.98–3.12)	0.059	1.19 (0.61–2.32)	0.611
Hyperlipidemia	19	1.64 (1–2.68)	0.051	0.98 (0.54–1.77)	0.939
Obesity	0	—	—		
**Medication**					
Hormonal medication	56	1.06 (0.73–1.55)	0.745	1.00 (0.68–1.48)	0.982

*Abbreviation: HR, hazard ratio; CI, confidence interval.

*Adjusted HR: adjusted for age, income, comorbidities and medication in Cox proportional hazards regression.

**Figure 2:**
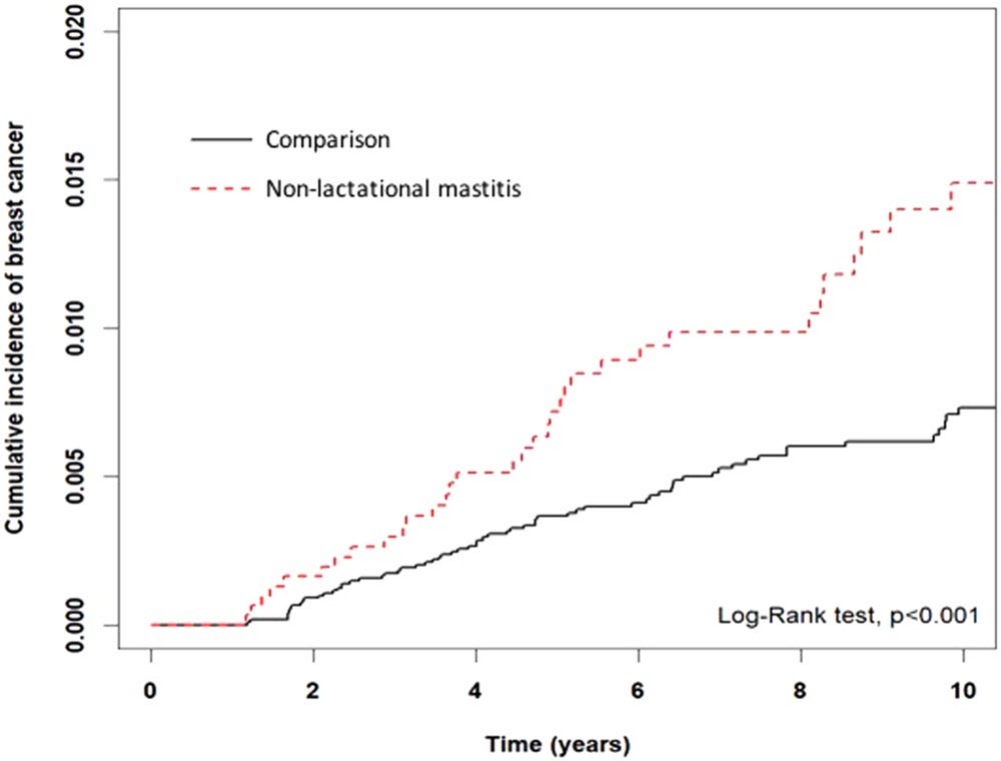
The cumulative incidences of breast cancer in women with non-lactational mastitis and the comparison group.

Figure 2 demonstrates that the cumulative incidence of breast cancer in women with non-lactational mastitis was higher than in the comparison group (p < 0.001). Table 3 presents the risk developing of breast cancer by incidence rate (IR) and HR after stratification by age, sex, comorbidities and hormonal medication. The overall IRs of breast cancer in the non-lactational mastitis and comparison groups were 14.79 and 7.57 per 10,000 person-years, respectively. Compared with women in the comparison group, women who had non-lactational mastitis before the age of 40 years (aHR = 2.22, 95% CI: 1.18–4.18) and at the age of 40 to 49 years of age (aHR = 2.00, 95% CI: 1.11–4.04) had a significantly higher risk of breast cancer. Non-lactational mastitis was a risk factor for breast cancer in women with lower SES (monthly income <USD$528, aHR = 2.60, 95% CI: 1.34–5.07) and in women who ever took hormonal medication (aHR = 1.96, 95% CI: 1.15–3.34). However, women with lactational mastitis did not exhibit a statistically significant risk of developing breast cancer, relative to women in the comparison group (Supplementary Tables S1–S3).

**Table 3:** Incidence rate, hazard ratio of breast cancer in different stratification.

	Comparison			Non-lactational mastitis			Non-lactational mastitis vs Comparison	
	n = 12364			n = 3091			Crude HR	Adjusted HR
	Event	Person years	IR	Event	Person years	IR	(95% CI)	(95% CI)
Overall	74	97722	7.57	37	25010	14.79	1.94 (1.31–2.87)**	1.94 (1.30–2.90)**
**Age at baseline**								
<40	26	62439	4.16	16	16168	9.90	2.36 (1.27–4.4)**	2.22 (1.18–4.18)*
40–49	28	21294	13.15	14	5245	26.69	2.00 (1.05–3.81)*	2.12 (1.11–4.04)*
50–59	14	9061	15.45	4	2454	16.30	1.03 (0.34–3.12)	1.28 (0.41–4.02)
≥60	6	4928	12.18	3	1143	26.25	2.21 (0.55–8.84)	1.80 (0.43–7.48)
**Monthly income (NT$)**								
0–15840	23	51975	4.43	15	12952	11.58	2.63 (1.37–5.04)**	2.60 (1.34–5.07)**
15841–28800	36	33946	10.61	16	8886	18.01	1.66 (0.92–3.00)	1.68 (0.93–3.06)
28801–45800	9	9125	9.86	5	2420	20.67	2.05 (0.69–6.12)	2.02 (0.66–6.16)
>45800	6	2676	22.42	1	753	13.29	0.59 (0.07–4.86)	0.53 (0.06–4.99)
**Baseline comorbidity**								
Schizophrenia	5	2227	22.46	1	622	16.07	0.67 (0.08–5.76)	0.84 (0.09–7.60)
Hypertension	12	9988	12.01	6	2997	20.02	1.60 (0.60–4.27)	1.63 (0.60–4.42)
Chronic obstructive pulmonary disease	11	9861	11.16	5	2766	18.07	1.61 (0.56–4.65)	1.39 (0.47–4.05)
Thyroid disease	5	7414	6.74	1	2144	4.66	0.67 (0.08–5.72)	0.61 (0.07–5.47)
Diabetes	10	7023	14.24	3	1913	15.68	1.08 (0.30–3.91)	1.05 (0.28–3.85)
Hyperlipidemia	14	11089	12.63	5	3099	16.13	1.24 (0.45–3.46)	1.16 (0.41–3.25)
Obesity	0	1064	0.00	0	320	0.00	—	—
**Medication**								
Hormonal medication	33	45423	7.27	23	16143	14.25	1.93 (1.13–3.28)*	1.96 (1.15–3.34)*

*Abbreviation: IR, incidence rates, per 10,000 person-years; HR, hazard ratio; CI, confidence interval.

*Adjusted HR: adjusted for age, income, comorbidities and medication in Cox proportional hazards regression.

*p-value < 0.05 *p-value < 0.01**.

Table 4 shows that the number of episodes of non-lactational mastitis affected the risk of breast cancer; most breast cancer cases were in the third tertile. There was a trend that the risk increased as the occurrence of non-lactational mastitis increased. The IR was 36.54 per 10,000 person-years (aHR = 5.50, 95% CI: 3.31–9.13) in the third tertile, compared to 7.57 per 10,000 person-years in the comparison group. The average time for development of breast cancer after the diagnosis of non-lactational mastitis was 6.03 years. The average numbers of episodes of non-lactational mastitis per year for women with and without breast cancer were 0.51 and 0.26, respectively (p = 0.005).

**Table 4:** Incidence rate and hazard ratio of breast cancer by the episode of non-lactational mastitis.

Non-lactational mastitis episode (mean per year)	Event	PY	IR	Crude HR (95% CI)	p-value	Adjusted HR (95% CI)	p-value
Comparison group (0)	74	97722	7.57	Ref.		Ref.	
First tertile (<0.14)	6	10732	5.59	0.68 (0.30–1.56)	0.36	0.69 (0.30–1.58)	0.38
Second tertile (0.14–0.27)	11	8805	12.49	1.61 (0.86–3.04)	0.14	1.68 (0.89–3.19)	0.11
Third tertile (>0.27)	20	5473	36.54	5.77 (3.50–9.53)	<0.001	5.50 (3.31–9.13)	<0.001

*Abbreviation: PY, person-years; IR, incidence rate, per 100,000 person-years; HR, hazard ratio; CI, confidence interval.

*Adjusted HR: adjusted for age, income, comorbidities and medication in Cox proportional hazards regression.

## Discussion

In this population-based study, we found that non-lactational mastitis occurred mostly in young women in Taiwan. Women who had non-lactational mastitis had a significantly higher risk of developing breast cancer than women who did not. Non-lactational mastitis was a risk factor of breast cancer among these three subgroups: women aged <50 years, women with lower socioeconomic status and women with hormonal medication. In addition, women who had more episodes of non-lactational mastitis had an increased risk of breast cancer development.

Consistent with the literature^27,28^, our findings demonstrated that middle aged and old aged women had higher risks of breast cancer than women aged <40 years (Table 2). In stratified analysis, there was a significantly increased risk of breast cancer in women with non-lactational mastitis aged <50. Women with non-lactational mastitis aged ≥50 years were also associated with an increased risk of breast cancer; however, this association was not statistically significant. This is potentially because non-lactational mastitis occurred mostly in younger women in Taiwan. In addition, the small numbers of women with non-lactational mastitis aged ≥50 years may be the reason for the lack of significance.

It is known that there is a small increase in the risk of breast cancer diagnoses in women taking oral contraceptives, as well as up to 10 years after discontinuation of oral contraceptives^29^. A higher percentage of women in our non-lactational mastitis group had a history of hormonal medication, relative to women in the comparison group; therefore, we stratified our analysis by hormonal medication history. Among women who had a history of hormonal medication, those who had non-lactational mastitis exhibited a significantly higher risk of breast cancer than those who did not have non-lactational mastitis. We could not attribute the increased risk of breast cancer in the women with non-lactational mastitis to hormonal medication.

Our results showed that the risk of breast cancer in women increased significantly as the occurrence of non-lactational mastitis increased. Non-lactational mastitis is known to be related to chronic inflammation secondary to infection-induced cytokines^30^, as well as inflammatory, infectious, hormonal and autoimmunity factors^31,32^. Reportedly, the presence of exogenous and endogenous pathogen-associated molecular patterns (PAMPs) (bacteria, viruses and fungi, as well as endogenous molecules released from injured or dying cells) can cause the activation of inflammatory pathways^33^. Chronic inflammation is further compounded by potentially malignant transformation^33–37^. Repeated occurrence of non-lactational mastitis in women may indicate that they are in a status of long-term exposure to these PAMPs that damages their breast and drives their breast cancer risk higher. However, further studies are needed to reveal the association of non-lactational mastitis with breast cancer. Our findings may serve as an epidemiological support for population-based observations.

The strength of this study is that it was a nationwide, population-based study. The NHI covers 99% of Taiwan’s population and has nearly complete medical records for the entire population. The results of this population-based cohort study are noteworthy, as non-lactational mastitis is not a very common disease, and the breast cancer incidence is approximately 188–194 per 100,000 women in Taiwan^38^; individual studies have been unable to reveal an association between non-lactational mastitis and breast cancer in women because they have been limited by either small sample size or cross-sectional design.

There were some limitations in this study. First, ICD codes were used to determine the diagnosis of mastitis. However, there is not a specific code to identify the entity and aetiology of non-lactational mastitis in the ICD-9 coding system. Second, this study did not identify when women stopped breastfeeding after delivery of a baby. In the literature, the median duration of breastfeeding is reportedly 7–12 months^39,40^. To reduce the misclassification of lactational mastitis as non-lactational mastitis, our cut-off for non-lactational mastitis was 1 year after giving birth to a baby. Third, residual and unmeasured confounding factors might exist; lifestyle-related variables (e.g. smoking and diet), family history and parity status are not recorded in the NHIRD. However, we were able to adjust for SES, comorbidities and hormonal medication, which are known risk factors for breast cancer. Finally, the follow-up period was short; however, the significance of non-lactational mastitis as a risk factor for breast cancer in the younger age group was dominant.

In summary, this study showed a significant risk of breast cancer in women with a history of non-lactational mastitis. Moreover, their risk of breast cancer was higher with increased frequency of non-lactational mastitis. The majority of the population in this study was in the younger age group (<40 years), and the follow-up period was short; therefore, further studies should be performed in a population with a wider age range and a longer follow-up period.

## Supplementary information

Risk of breast cancer in women with non-lactational mastitis

## Data Availability

The dataset is not available for public access, but is available from the Taiwan National Health Insurance Department on reasonable request.

## References

[1] Bray F (2018). Global cancer statistics 2018: GLOBOCAN estimates of incidence and mortality worldwide for 36 cancers in 185 countries. CA: a cancer journal for clinicians.

[2] Siegel RL, Miller KD, Jemal A (2018). Cancer statistics, 2018. CA: a cancer journal for clinicians.

[3] Chen L, Linden HM, Anderson BO, Li CI (2014). Trends in 5-year survival rates among breast cancer patients by hormone receptor status and stage. Breast cancer research and treatment.

[4] ACOG Committee Opinion No. 361 (2007). Breastfeeding: maternal and infant aspects. Obstetrics and gynecology.

[5] Al-Khaffaf B, Knox F, Bundred NJ (2008). Idiopathic granulomatous mastitis: a 25-year experience. Journal of the American College of Surgeons.

[6] Abdelhadi MS, Bukharie HA (2005). Breast infections in non-lactating women. Journal of family & community medicine.

[7] Bouton ME, Jayaram L, O’Neill PJ, Hsu CH, Komenaka IK (2015). Management of idiopathic granulomatous mastitis with observation. American journal of surgery.

[8] Zhang Y, Zhou Y, Mao F, Guan J, Sun Q (2018). Clinical characteristics, classification and surgical treatment of periductal mastitis. Journal of thoracic disease.

[9] Cordon-Cardo C, Prives C (1999). At the crossroads of inflammation and tumorigenesis. The Journal of experimental medicine.

[10] Coussens LM, Werb Z (2002). Inflammation and cancer. Nature.

[11] Karin M, Lawrence T, Nizet V (2006). Innate immunity gone awry: linking microbial infections to chronic inflammation and cancer. Cell.

[12] Kuper H, Adami HO, Trichopoulos D (2000). Infections as a major preventable cause of human cancer. Journal of internal medicine.

[13] Al Bakir I, Curtius K, Graham TA (2018). From Colitis to Cancer: An Evolutionary Trajectory That Merges Maths and Biology. Frontiers in immunology.

[14] Cai H (2015). Cholelithiasis and the risk of intrahepatic cholangiocarcinoma: a meta-analysis of observational studies. BMC cancer.

[15] Stinton LM, Shaffer EA (2012). Epidemiology of gallbladder disease: cholelithiasis and cancer. Gut and liver.

[16] Zhuo C, Triplett PT (2018). Association of Schizophrenia With the Risk of Breast Cancer Incidence: A Meta-analysis. JAMA psychiatry.

[17] Han H (2017). Hypertension and breast cancer risk: a systematic review and meta-analysis. Scientific reports.

[18] Ho CH, Chen YC, Wang JJ, Liao KM (2017). Incidence and relative risk for developing cancer among patients with COPD: a nationwide cohort study in Taiwan. BMJ open.

[19] Sogaard M (2016). Hypothyroidism and hyperthyroidism and breast cancer risk: a nationwide cohort study. European journal of endocrinology.

[20] Hardefeldt PJ, Eslick GD, Edirimanne S (2012). Benign thyroid disease is associated with breast cancer: a meta-analysis. Breast cancer research and treatment.

[21] Tsilidis KK, Kasimis JC, Lopez DS, Ntzani EE, Ioannidis JP (2015). Type 2 diabetes and cancer: umbrella review of meta-analyses of observational studies. BMJ (Clinical research ed.).

[22] Nelson ER (2013). 27-Hydroxycholesterol links hypercholesterolemia and breast cancer pathophysiology. Science (New York, N.Y.).

[23] Kyrgiou M (2017). Adiposity and cancer at major anatomical sites: umbrella review of the literature. BMJ (Clinical research ed.).

[24] Austin PC (2014). A comparison of 12 algorithms for matching on the propensity score. Statistics in medicine.

[25] Austin PC (2011). An Introduction to Propensity Score Methods for Reducing the Effects of Confounding in Observational Studies. Multivariate behavioral research.

[26] Austin PC (2009). Balance diagnostics for comparing the distribution of baseline covariates between treatment groups in propensity-score matched samples. Statistics in medicine.

[27] Muss HB (2011). Coming of age: breast cancer in seniors. The oncologist.

[28] Smigal C (2006). Trends in breast cancer by race and ethnicity: update 2006. CA: a cancer journal for clinicians.

[29] Breast cancer and hormonal contraceptives (1996). collaborative reanalysis of individual data on 53 297 women with breast cancer and 100 239 women without breast cancer from 54 epidemiological studies. Lancet (London, England).

[30] Liu L (2017). Periductal Mastitis: An Inflammatory Disease Related to Bacterial Infection and Consequent Immune Responses?. Mediators of inflammation.

[31] Sheybani F, Sarvghad M, Naderi HR, Gharib M (2015). Treatment for and clinical characteristics of granulomatous mastitis. Obstetrics and gynecology.

[32] Gopalakrishnan Nair C, Hiran, Jacob P, Menon RR (2015). Misha Inflammatory diseases of the non-lactating female breasts. International journal of surgery (London, England).

[33] Bhatelia K, Singh K, Singh R (2014). TLRs: linking inflammation and breast cancer. Cellular signalling.

[34] Jukkola-Vuorinen A (2009). Toll-like receptor-9 expression is inversely correlated with estrogen receptor status in breast cancer. Journal of innate immunity.

[35] Amarante MK (2012). Toll-like receptor 3: implications for proinflammatory microenvironment in human breast cancer. Molecular biology reports.

[36] Ehsan N (2013). Significant correlation of TLR4 expression with the clinicopathological features of invasive ductal carcinoma of the breast. Tumour biology: the journal of the International Society for Oncodevelopmental Biology and Medicine.

[37] Liao SJ (2012). Triggering of Toll-like receptor 4 on metastatic breast cancer cells promotes alphavbeta3-mediated adhesion and invasive migration. Breast cancer research and treatment.

[38] https://www.hpa.gov.tw/Pages/Detail.aspx?nodeid=205&pid=1124 accessed on February 19 (2019).

[39] Waldenstrom U, Aarts C (2004). Duration of breastfeeding and breastfeeding problems in relation to length of postpartum stay: a longitudinal cohort study of a national Swedish sample. Acta paediatrica (Oslo, Norway: 1992).

[40] Toryiama ATM (2017). Breastfeeding: what changed after a decade?1. Revista latino-americana de enfermagem.

