# Supplementary material for: Risk of breast cancer in women with non-lactational mastitis
Source: Sci Rep. 2019 Oct 30;9:15587. doi: 10.1038/s41598-019-52046-3 (PMC6821708; doi:10.1038/s41598-019-52046-3)
Supplement: Supplementary file 1 — Risk of breast cancer in women with non-lactational mastitis [file 41598_2019_52046_MOESM1_ESM.pdf]

Risk of breast cancer in women with non-lactational mastitis

Chun-Ming Chang [a,d], Mei-Chen Lin [b,e], Wen-Yao Yin [c,d*]

a. Department of General Surgery, Hualien Tzu Chi Hospital, Buddhist Tzu Chi Medical Foundation, Hualien, Taiwan
b. Management Office for Health Data, China Medical University Hospital, Taichung, Taiwan
c. Department of General Surgery, Dalin Tzu Chi Hospital, Buddhist Tzu Chi Medical Foundation, Chiayi, Taiwan
d. College of Medicine, Tzu Chi University, Hualien, Taiwan
e. College of Medicine, China Medical University, Taichung, Taiwan

***Corresponding Author**

Wen-Yao Yin

Department of General Surgery, Dalin Tzu Chi Hospital, Buddhist Tzu Chi Medical Foundation, Chiayi, Taiwan

No. 2, Minsheng Rd., Dalin Township, Chiayi County 622, Taiwan

E-mail address: wenyao4748@gmail.com

Supplementary information

Table S1. Demographic characteristics of lactational mastitis women and the comparison group

	Total n=3072 n	Comparison n=1536 n (%) / mean ± SD	Lactational mastitis n=1536 n (%) / mean ± SD	Standardized mean difference[§]
**Age at baseline‡**				
<30	1626	823 (53.6)	803 (52.3)	0.085
≥30	1446	713 (46.4)	733 (47.7)	0.085
**Monthly income (NT$)**				
0-15840	2517	1259 (82)	1258 (81.9)	0.002
15841-28800	447	232 (15.1)	215 (14)	0.031
28801-45800	85	37 (2.4)	48 (3.1)	0.044
>45800	23	8 (0.5)	15 (1)	0.053
**Baseline comorbidity**				0.000
Schizophrenia	4	2 (0.1)	2 (0.1)	0.025
Hypertension	34	19 (1.2)	15 (1)	0.020
Chronic obstructive pulmonary disease	218	105 (6.8)	113 (7.4)	0.013
Thyroid disease	223	114 (7.4)	109 (7.1)	0.012
Diabetes	79	38 (2.5)	41 (2.7)	0.012
Hyperlipidemia	66	32 (2.1)	34 (2.2)	0.009
Obesity	16	7 (0.5)	9 (0.6)	0.018
**Medication**				
Hormonal medication	2237	1100 (71.6)	1137 (74)	0.054

Abbreviation: SD, standard deviation.

‡Student's t test

[§]A standardized mean difference of <0.1 indicates a negligible difference between the two cohorts.

Table S2. Cox proportional hazards regression measured hazard ratio of breast cancer

	Event	Crude		Adjusted	
	(n=14)	HR (95% CI)	p-value	HR (95% CI)	p-value
**Lactational mastitis**					
No	6	Ref.		Ref.	
Yes	8	1.35(0.47-3.9)	0.576	1.46(0.5-4.2)	0.488
**Age at baseline**					
<30	1	Ref.		Ref.	
≥30	13	17.46(2.28-133.7)	0.006	23.36(3-182)	0.003
**Monthly income (NT$)**					
0-15840	11	Ref.		Ref.	
15841-28800	3	0.93(0.25-3.42)	0.918	0.47(0.13-1.78)	0.268
28801-45800	0	0(0-.)	0.993	0(0-.)	0.997
>45800	0	0(0-.)	0.996	0(0-.)	0.998
**Baseline comorbidity**					
Schizophrenia	0	0(0-.)	0.995	0(0-.)	1.000
Hypertension	0	0(0-.)	0.994	0(0-.)	0.998
Chronic obstructive pulmonary disease	0	0(0-.)	0.991	0(0-.)	0.996
Thyroid disease	0	0(0-.)	0.990	0(0-.)	0.996
Diabetes	0	0(0-.)	0.990	0(0-.)	0.997
Hyperlipidemia	0	0(0-.)	0.992	0(0-.)	0.998
Obesity	0	0(0-.)	0.992	0(0-.)	0.999
**Medication**					
Hormonal medication	9	0.78(0.26-2.32)	0.651	0.76(0.25-2.28)	0.625

*Abbreviation: HR, hazard ratio; CI, confidence interval.

*Adjusted HR: adjusted for age, income, comorbidities and medication in Cox proportional hazards regression.

Table S3. Incidence rate, hazard ratio of breast cancer in different stratification

	Comparison n=1536			Lactational mastitis n=1536			Lactational mastitis vs Comparison	
	Event	Person years	IR	Event	Person years	IR	Crude HR (95% CI)	Adjusted HR (95% CI)
**Overall**	6	10911	5.50	8	10788	7.42	1.35(0.47-3.90)	1.46(0.50-4.20)
**Age at baseline**								
<30	0	6136	0.00	1	6088	1.64	-	-
≥30	6	4775	12.56	7	4700	14.89	1.22(0.41-3.63)	1.26(0.42-3.77)
**Monthly income (NT$)**								
0-15840	6	8225	7.29	5	8194	6.10	0.83(0.25-2.73)	0.94(0.29-3.11)
15841-28800	0	2269	0.00	3	2002	14.99	-	-
28801-45800	0	331	0.00	0	440	0.00	-	-
>45800	0	86	0.00	0	152	0.00	-	-
**Baseline comorbidity**								
Schizophrenia	0	5	0.00	0	12	0.00	-	-
Hypertension	0	153	0.00	0	98	0.00	-	-
Chronic obstructive pulmonary disease	0	696	0.00	0	675	0.00	-	-
Thyroid disease	0	796	0.00	0	715	0.00	-	-
Diabetes	0	325	0.00	0	309	0.00	-	-
Hyperlipidemia	0	212	0.00	0	201	0.00	-	-
Obesity	0	37	0.00	0	59	0.00	-	-
**Medication**								
Hormonal medication	3	7619	3.94	6	7683	7.81	2.01(0.50-8.05)	2.09(0.52-8.37)

*Abbreviation: IR, incidence rates, per 10,000 person-years; HR, hazard ratio; CI, confidence interval.

*Adjusted HR: adjusted for age, income, comorbidities and medication in Cox proportional hazards regression.
